# Treatment of Kimura's disease with oral corticosteroid and methotrexate^☆^^☆☆^

**DOI:** 10.1016/j.abd.2019.03.006

**Published:** 2019-12-18

**Authors:** Han Ma

**Affiliations:** Department of Dermatology, the Fifth Affiliated Hospital, Sun Yat-sen University, Zhuhai, Guangdong, China

Dear Editor,

Kimura's disease (KD) was initially described by Kim and Szeto in 1937, and became better known after a systematic description provided by Kimura as a chronic inflammatory disease.^1^ Most cases reported occurred in Asian men between 20 and 30 years of age.^2^ Therapeutic modalities for KD include surgical excision, radiotherapy, and various immunomodulating agents, such as oral corticosteroids, cyclosporine, leflunomide, and mycophenolate mofetil.^3^ We report a case of KD with an excellent and sustained response to oral corticosteroid and intravenous methotrexate. A 51-year-old man presented with a history of fullness of the bilateral upper eyelids and a similar swelling in the bilateral parotid regions for seven years (Fig. 1); itching or pain symptoms. Physical examination revealed soft, pendular, non-tender mass lesions on both lateral upper eyelids, resulting in mechanical ptosis. The remainder of the ocular examination was within normal limits. His past medical history was unremarkable. Complete rheumatologic and immunologic workup was performed. Complete blood count showed the total number of white blood cells was 8.3 × 10^9^/L, neutrophils 4.35 × 10^9^/L (accounting for 52.4%), lymphocytes 2.50 × 10^9^/L (accounting for 30.1%), and eosinophils 1.01 × 10^9^/L (accounting for 12.2%). Serum IgE was 205 IU/mL (normal, <100). Remaining laboratory results were normal. Computed tomography scan revealed soft-tissue lesions involving both the upper eyelid and parotid regions. A post-contrast study showed intense homogeneous enhancement on delayed scans (Fig. 2). Histopathology of the lesion excised from the left upper eyelid showed lymphoid tissue hyperplasia, with lymphoid nodules containing germinal centers that were scattered in the dermis and subcutaneous tissue, with scattered eosinophilic infiltration (Fig. 3). Based on the clinical manifestations and histopathological features, KD was then diagnosed. The therapeutic regimen comprised a tapering dose of oral prednisone (initial dose 40 mg/d) and intravenous methotrexate at 15 mg/week for two months. The patient had complete resolution after treatment and there was no recurrence in the next two years of follow-up. KD is a chronic inflammatory disease that manifests as a triad of subcutaneous nodules in the head and neck region, peripheral blood eosinophilia, and elevated serum IgE.^3^ It may also involve extracutaneous sites, such as regional lymph nodes, major salivary glands, and the kidneys. However, renal involvement is not uncommon and most frequently results in nephritic syndrome.^4^ The patient presented all the three typical elements to fulfill the diagnostic criteria and both sides of salivary glands had been involved. Thus, KD was the first diagnosis considered. This disease must be distinguished from angiolymphoid hyperplasia with eosinophilia (ALHE) because of several overlapping clinical and histologic features. KD occurs mainly in young men of Asian descent with one or multiple asymptomatic masses involving the subcutaneous tissue and salivary glands. It is often accompanied by regional lymph node involvement, peripheral blood eosinophilia, and elevated IgE. In contrast, ALHE occurs predominantly in middle-aged women, presenting with multiple small papules or erythematous nodules associated with itching.^1^ In the histopathologic features, KD displays the presence of numerous lymphoid follicles and the absence of irregular, dilated blood vessels,^2^ just like what was observed in this case. The pathogenesis of KD remains unknown, but allergy, atopy, autoimmunity, and parasite infestation are considered possible risk factors.^3^ Previous studies have found increased levels of interleukin-4, interleukin-5, and interleukin-13 in the peripheral blood of affected individuals, suggesting a role for type 2 T-helper cytokines.^5^ Therapeutic methods reported in the literature are heterogeneous, but surgical excision and oral corticosteroids represent the most frequently used strategies.^3^ To avoid recurrence in the course of tapering steroids, various immunomodulating agents should be added in the treatment plan. Leflunomide and mycophenolate mofetil have shown promise effective in some reported cases.^3^ But the two drugs are still expensive, so we chose methotrexate as the combined drug, which exhibits immunomodulatory effects in a similar fashion by inhibiting *de novo* purine synthesis *via* inosine monophosphate dehydrogenase. Although recurrence is very common, it did not occurred in the present patient within the next two years of follow-up. The author feels that methotrexate may be a promising therapy for KD.

**Figure 1:**
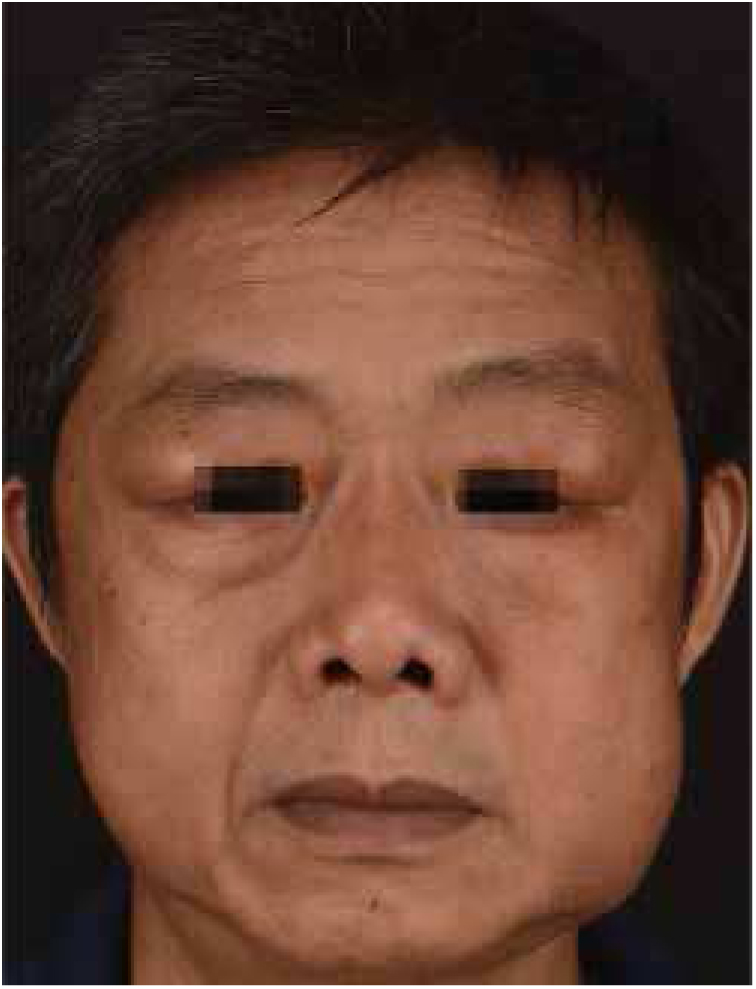
Fullness of the bilateral upper eyelids and swelling in the bilateral parotid regions.

**Figure 2:**
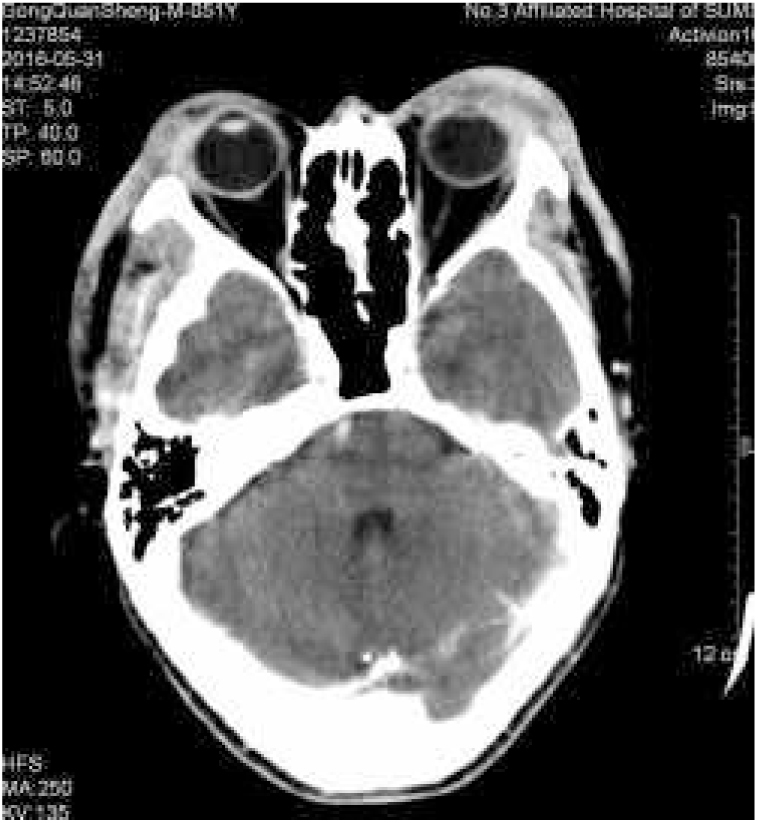
Soft-tissue lesions involving both the upper eyelid and parotid regions.

**Figure 3:**
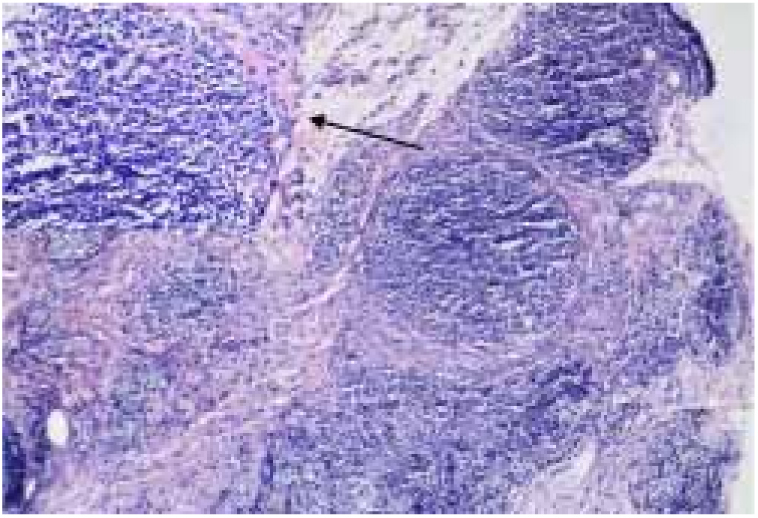
Nodular lymphocytic infiltrate with germinal centers involving the dermis and subcutaneous tissue, and reactive germinal centers surrounded by small mature lymphocytes and eosinophils (arrow) (Hematoxylin & eosin, ×100).

## Financial support

None declared.

## Author's contribution

Han Ma: Approval of the final version of the manuscript; elaboration and writing of the manuscript.

## Conflicts of interest

None declared.
